# Occurrence and distribution of *Salmonella* serovars in carcasses and foods in southern Italy: Eleven-year monitoring (2011–2021)

**DOI:** 10.3389/fmicb.2022.1005035

**Published:** 2022-10-06

**Authors:** Maria Francesca Peruzy, Yolande Thérèse Rose Proroga, Federico Capuano, Andrea Mancusi, Angela Michela Immacolata Montone, Daniela Cristiano, Anna Balestrieri, Nicoletta Murru

**Affiliations:** ^1^Department of Veterinary Medicine and Animal Production, University of Naples Federico II, Naples, Italy; ^2^Department of Food Microbiology, Istituto Zooprofilattico Sperimentale del Mezzogiorno, Portici, Italy; ^3^Task Force on Microbiome Studies, University of Naples Federico II, Naples, Italy

**Keywords:** *Salmonella* occurrence in foods, *Salmonella* occurrence in carcasses of food-producing animals, *Salmonella* serovars, *S*. Infantis, monophasic *S*. Typhimurium, *S*. Typhimurium

## Abstract

*Salmonella* is one of the most common agents of foodborne illness. The genus *Salmonella* includes two species (*Salmonella bongori* and *S. enterica*) and six subspecies (*enterica* I, *salamae* II, *arizonae* IIIa, *diarizonae* IIIb, *houtenae* IV, and *indica* VI), each of which contains multiple serotypes associated with animal and human infections. The aim of the study was to evaluate the presence of *Salmonella* spp. in carcasses of food-producing animals and foods in southern Italy and the serovar distribution among different sources. From 2011 to 2021, a total of 12,246 foods and 982 samples from animal carcasses were collected and analyzed. The overall percentage of positive samples was 5.84% (*N* = 773) and a significant increase in prevalence was observed by comparing the years 2011–2015 (257, 3.27%) and 2016–2021 (516, 9.61%; *p* < 0.05). The highest percentage of positive food samples was observed in “Meat and Meat Products” (*N* = 327/2,438, 13.41%) followed by “Fish and fishery products” (*N* = 115/1,915, 6.01%). In carcasses, the highest percentage of positive samples was reported from broilers (*N* = 42/81, 51.85%) followed by buffalo (*N =* 50/101, 49.50%) and pork (*N =* 140/380, 36.84%). After typing, the isolates were assigned to the species *S. enterica* and to the subspecies: *enterica* (*N =* 760, 98.32%), *diarizonae* (*N =* 8, 1.03%), *salamae* (*N =* 3, 0.39%) and *houtenae* (*N =* 2, 0.26%). *S.* Infantis was the most frequently detected (*N =* 177, 24.76%), followed by *S.* Derby (*N =* 77, 10.77%), monophasic *S*. Typhimurium (*N =* 63, 8.81%), *S*. Typhimurium (*N =* 54, 7.55%), and *S.* Rissen (*N =* 47, 6.57%). By comparing the sampling period 2011–2015 with that of 2016–2021, an increase in the prevalence of *S.* Infantis and monophasic *S*. Typhimurium and a decrease of *S*. Typhimurium were recorded (*p* < 0.05). Thus, present data suggest that, despite the implementation of national and European control strategies to protect against *Salmonella*, the prevalence of this pathogen in southern Italy is still increasing and a change of national control programs to protect against *Salmonella* are necessary.

## Introduction

Acute gastroenteritis remains a significant cause of morbidity and mortality worldwide and approximately one-third of these infections are transmitted through food (Besser, 2018). According to the most recent report of the World Health Organization (WHO) in 2010, 31 foodborne hazards resulted in over 600 million illnesses and 420,000 deaths worldwide (Li et al., 2019). Among 31 pathogens, *Salmonella* is one of the most common agents of foodborne illness causing over 76 million diarrhea-associated diseases a year in the world (Chlebicz and Śliżewska, 2018; Popa and Popa, 2021). In Europe, Salmonellosis was the second most reported zoonosis in 2020 with 52,702 cases of illness and was an important cause of foodborne outbreaks mainly associated with the consumption of eggs and eggs products, bakery products and pig meat and products thereof [EFSA and ECDC (European Food Safety Authority and European Centre for Disease Prevention and Control), 2021]. In Italy, with more than 3,000 human cases reported per year [EFSA and ECDC (European Food Safety Authority and European Centre for Disease Prevention and Control), 2021], Salmonellosis is the most common foodborne infection (Leati et al., 2021). The genus *Salmonella* includes two species, *Salmonella* (*S.*) *bongori* and *S. enterica*, and six subspecies: *enterica* I, *salamae* II, *arizonae* IIIa, *diarizonae* IIIb, *houtenae* IV, and *indica* VI. The subspecies *enterica* is principally associated with warm-blooded animals while *Salmonella* non-*enterica* subspecies are commonly in cold-blooded animals (Hounmanou et al., 2022). The subspecies *enterica*, according to its surface antigens (O and H), can be divided into 1,547 serovars of which 99% are associated with animal and human infections (Ferrari et al., 2019). In 2020, the top five *Salmonella* serovars involved in human infections in Europe were *S.* Enteritidis, *S*. Typhimurium, monophasic *S*. Typhimurium (1,4, [5],12:i:-), *S.* Infantis, and *S.* Derby [EFSA and ECDC (European Food Safety Authority and European Centre for Disease Prevention and Control), 2021]. In Italy however, *S.* Napoli ranks among the top serovars causing human infections even though it is relatively uncommon in other European countries (Leati et al., 2021).

Human infections caused by serovars belonging to the subspecies *S. enterica* are generally self-limiting gastroenteritis and do not require antimicrobial treatment (Peruzy et al., 2020; Sodagari et al., 2020). These serovars recognize the gastrointestinal tract of a wide range of domestic and wild animals as the reservoir and can reach humans following direct contact with infected animals or *via* the consumption of infected products or foods prepared without following proper hygiene procedures. Eggs and egg products represent the main source of human infection followed by meat, whose contamination mainly results from improper slaughter hygiene (Chlebicz and Śliżewska, 2018).

The worldwide epidemiology of *Salmonella* serovars is complex in terms of its distribution and transmission (Ferrari et al., 2019). The prevalence of *Salmonella* and the distribution of serovars in food matrices may also vary according to countries as it is influenced both by culture and food production practices as well as by geographic location (Ferrari et al., 2019; Cohn et al., 2021). Therefore it is essential to monitor the possible contamination routes for humans. At present, long-term studies on the occurrence and serovar distribution of *Salmonella* in non-human sources are limited in Europe The collection of updated data is essential for the development of regional and serovar-specific intervention and control programmes.

For this reason, the present work aimed to evaluate the changes between 2011 and 2021 of the prevalence of *Salmonella* in carcasses of food-producing animals and foods in two regions (Campania and Calabria) of southern Italy and the serovar distribution among the different sources for the prompt identification of potential risks for public health.

## Materials and methods

### Sampling

From 2011 to 2021, a total of 12,246 foods [definition of food according to the Regulation (EC) No 178/2002] and 982 samples from animal carcasses (Table 1; Supplementary Table 1) were collected in Campania (N. of samples = 6,378) and Calabria (N. of samples = 6,834) in southern Italy and analyzed to detect *Salmonella* spp. No information regarding the geographical location of collection was recorded for 16 samples.

**Table 1:** Number (N) of samples collected in the context of official controls provided by Regulation (EC) No 2073/2005 and during self-monitoring from public or private enterprises and number (N) of *Salmonella*-positive samples grouped by source and years (2011–2015 vs. 2016–2021).

	2011–2015			2016–2021
Matrix
	*N* sampled units	Positive (*N*, %)		*N* sampled units	Positive (*N*, %)
**Milk and milk products** ^*^	**2,374**	**7**	**0.29**	**901**	**9**
Processed milk	28	0	0	30	1
Raw milk	89	4	4.49	54	6
Dairy products	2,257	3	0.13	817	2
**Meat and meat products** ^*^	**1,356**	**131**	**9.73**	**1,082**	**196**
Meat and meat products from bovine	129	12	9.30	187	17
Meat and meat products from buffalo	4	2	50.00	0	
Meat and meat products from equine	2	0	0	0	
Meat and meat products from ovine	2	0	0	3	0
Meat and meat products from pork	292	69	23.63	211	40
Meat and meat products from broiler^*^	147	21	14.29	269	110
Meat and meat products from rabbit	2	0	0	0	
Meat and meat products from Turkey	21	12	57.14	15	5
Meat and meat products from wild boar	3	3	100.00	2	1
MMP from broiler and Turkey	3	3	100.00	18	18
MMP from pork and bovine	0			1	1
MMP from broiler, Turkey and pork	0			2	2
NI	751	9	1.20	374	2
**Fish and fishery products** ^*^	**980**	**43**	**4.39**	**935**	**72**
Bivalve mollusks*	619	26	4.20	795	71
Cephalopod mollusks	37	3	8.11	21	0
Mollusks	14	13	92.86	1	0
Crustaceans	15	0	0	8	1
Others	295	1	0.34	110	0
**Fruits and vegetables and juices**	**597**	**3**	**0.50**	**148**	**3**
**Eggs and egg products**	**332**	**5**	**1.51**	**194**	**3**
**Bakery products**	**185**	**0**	**0**	**275**	**1**
**Infant formulae**	**141**	**0**	**0**	**110**	**0**
**Seeds (sprouted seeds)**	**59**	**11**	**18.64**	**35**	**3**
**Cereals**	**39**	**1**	**2.56**	**38**	**0**
**Spices and herbs**	**8**	**1**	**12.50**	**31**	**0**
**Soft drink**	**18**	**0**		**7**	**0**
**Pasta**	**5**	**0**		**19**	**0**
**Potable water**	**15**	**0**	**0**	**4**	**0**
**Honey**	**4**	**0**	**0**	**5**	**0**
**Other processed food products and prepared dishes**	**1,279**	**0**	**0**	**1,070**	**2**
**Carcass** ^*^	**468**	**55**	**11.75**	**514**	**227**
Carcass of bovine^*^	210	2	0.95	132	35
Carcass of buffalo^*^	74	23	31.08	27	27
Carcass of goat	5	1	20.00	0	
Carcass of ovine	11	1	9.09	2	1
Carcass of pork^*^	99	9	9.09	281	131
Carcass of broiler	34	15	44.12	47	27
Carcass of wild boar	4	4	100.00	6	6
NI	31	0	0	19	0
**All** ^*^	**7,860**	**257**	**3.27**	**5,368**	**516**

The asterisk (*) indicates the significant differences between the two sampling periods (*p* <0.05). NI, no information on animal’s species. Bold values mean food samples were grouped into: Milk and milk products, Meat and Meat Products, Fish and fishery products =, Fruits, vegetables and juices, Eggs and egg products, Bakery products, Infant formulae, Seeds (sprouted seeds), Cereals, Spices and herbs and, Soft drinks, Pasta, Potable water, Honey, other processed food products, prepared dishes and carcasses.

Food samples were collected in the context of official controls provided by Regulation (EC) No 2073/2005 on microbiological food safety criteria and during own-check sampling from public or private enterprises. Food samples were grouped into 15 categories: Milk and milk products = 3,275, Meat and Meat Products = 2,438, Fish and fishery products = 1915, Fruits, vegetables and juices = 745, Eggs and egg products = 526, Bakery products = 460, Infant formulae = 251, Seeds (sprouted seeds) = 94, Cereals = 77, Herbs and spices = 39, Soft drinks = 25, Pasta = 24, Potable water = 19, Honey = 9 and other processed food products and prepared dishes = 2,349 (Table 1). The carcass samples were collected at different slaughterhouses from pork (*N =* 380), bovine (*N =* 342), buffalo (*N =* 101), broilers (*N =* 81), ovine (*N =* 13), wild boar (*N =* 10) and goat (*N =* 5; Table 1). For the broilers, 25 *g* of neck skin were aseptically collected from each carcass. The samples from carcasses belonging to the other animal species were collected using cellulose sponges (WPB01245WA, Sigma-Aldrich; non-destructive method) prehydrated with 10 ml sterilized peptone water following the ISO 176604/2015 in the context of official controls provided by the Regulation (EC) No 2073/2005 on microbiological process hygiene criteria and during self-monitoring controls.

Samples were transported at 4°C to the laboratory and processed within 1 h after sampling.

### Detection and serotyping analysis

Twenty-five g/ml portions of each sample and 10 ml of sponge eluates were homogenized, respectively, in 225 ml and 90 ml (1:10 (W/W)) of buffer peptone water (BPW, CM0509, Oxoid) and incubated at 37°C for 18-h. Subsequently, 0.1 and 1.0 ml of the incubated homogenates were transferred into Rappaport Vassiliadis broth (RVS, CM0669, Oxoid) and Muller Kaufman broth (MK, CM1048, Oxoid) and incubated at 41.5°C for 24-h and 37°C for 24-h, respectively. Then the enrichments were streaked into xylose-lysinedesoxycholate agar (XLD, CM0469, Oxoid) and *Salmonella* chromogenic agar base (CM1007, Oxoid) with salmonella selective supplement (SR0194, Oxoid) and incubated at 37°C for 24-h. Presumptive *Salmonella* colonies were biochemically identified through API 20 E. Afterwards, the isolates were serotyped at the Campania Region *Salmonella* Typing Center (Department of Food Microbiology, Istituto Zooprofilattico Sperimentale del Mezzogiorno, Portici, NA, Italy) following the Kaufmann–White scheme.

### Statistical analysis

The differences in the occurrence of *Salmonella* spp. in the different sources, different investigated areas (Campania and Calabria), and different sampling periods (2011–2015 vs. 2016–2021) were assessed using the chi-square test. A probability value of less than 0.05 (*p* < 0.05) was defined as statistically significant.

## Results

### Prevalence of *Salmonella* spp. in different matrices

A total of 13,228 samples were analyzed for *Salmonella* spp. from 2011 to 2021. Regardless of the nature of the sample, the overall percentage of positive samples was 5.84% (*N =* 773; Table 1). Of the two investigated areas, Campania showed the highest percentage of positive samples (Campania = 574, 9.00% *vs* Calabria = 184, 2.70%; *p* < 0.05). No information regarding the geographical location of collection was recorded for 15 positive samples.

The percentage of positive samples ranged from 2.46% in 2014 to 18.56 in 2021 (Supplementary Table 1). The comparison between the sampling periods 2011–2015 and 2016–2021 showed an increase in the prevalence of *Salmonella* spp. (2011–2015 = 257, 3.27% vs. 2016–2021 = 516, 9.61%; Table 1). The occurrence of *Salmonella* spp. between the two sampling periods showed a significant difference (*p* < 0.05) observed both in Campania (2011–2015 = 190, 4.83% vs. 2016–2021 = 384, 15.73%) and Calabria (2011–2015 = 65, 1.66% *vs* 2016–2021 = 119, 4.08%; *p* < 0.05).

The overall percentage of food positive samples was 2.73% (*N =* 202) in the years 2011–2015 and 5.95% (289) in the years 2016–2021 (*p* < 0.05).

By source, the highest percentage of *Salmonella*-positive samples among the food analysed was reported in seed and sprouted seeds (*N =* 14/94, 14.89%) followed by “Meat and Meat Products” (*N =* 327/2,438, 13.41%; *p* < 0.05) and “Fish and fishery products” (*N =* 115/1,915, 6.01%; Table 1).

Within the category of “Meat and Meat Products,” excluding those with a limited number of collected samples, the majority of the isolates were obtained from turkeys (*N =* 17/36, 47.22%), followed by broilers (*N =* 131/416, 31.49%). This latter category showed a significant increase in the prevalence between the two sampling periods (2011–2015 vs. 2016–2021; *p* < 0.05). Within the “Fish and fishery products,” a significant increase in *Salmonella* isolation between the two sampling periods was observed in bivalve mollusks (*p* < 0.05; Table 1).

The overall percentage of carcasses showing positive samples was 28.72% (*N =* 282/982); within this category and excluding those with a limited number of collected samples, the highest percentage of positive samples was reported from broilers (*N =* 42/81, 51.85%) followed by buffalo (*N =* 50/101, 49.50%) and pork (*N =* 140/380, 36.84%; Table 1). In addition, the pork, buffalo and bovine carcasses showed a significant increase in the occurrence of *Salmonella* between the two sampling periods (*p* < 0.05; Table 1).

### Serovars distribution between different matrices

The isolates were assigned to the species *Salmonella enterica* and to the subspecies: *enterica* (*N =* 760, 98.32%), *diarizonae* (*N =* 8, 1.03%), *salamae* (*N =* 3, 0.39%) and *houtenae* (*N =* 2, 0.26%). Among the subspecies *enterica*, serotyping identified a total of 79 serovars (2011–2015 = 55 serovars, 2016–2021 = 51; Supplementary Table 2). No information regarding the serovar was recorded for 45 isolates.

**Figure 1:**
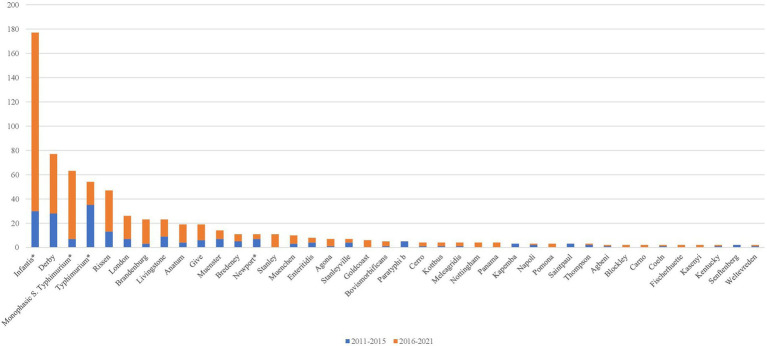
Number of *Salmonella* serovars within the subspecies *enterica* isolated with an occurrence >1 from 2011 to 2021. The asterisk (^*^) indicates the significant differences between the two sampling periods (2011–2015 vs. 2016–2021; *p* < 0.05).

During the entire study period (2011–2021), *S.* Infantis was the most frequently detected (*N =* 177, 24.76%) among the identified serovars (*N =* 715), followed by *S.* Derby (*N =* 77, 10.77%), monophasic *S*. Typhimurium (*N =* 63, 8.81%), *S*. Typhimurium (*N =* 54, 7.55%), and *S.* Rissen (*N =* 47, 6.57%; Figure 1). By comparing the sampling period 2011–2015 with 2016–2021, an increase in the prevalence of *S.* Infantis and monophasic *S*. Typhimurium and a decrease of *S*. Typhimurium were recorded. The observed differences were statistically significant (*p* <0.05; Figure 1).

**Figure 2:**
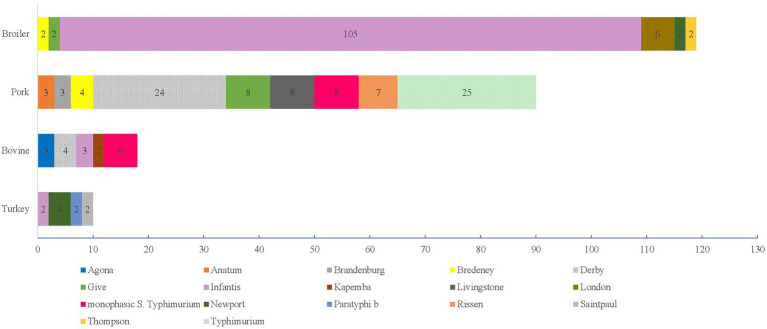
Number of *Salmonella* serovars within the subspecies *enterica* with an occurrence >1 from 2011 to 2021 isolated from Broilers, Pork, Bovine and Turkey.

**Figure 3:**
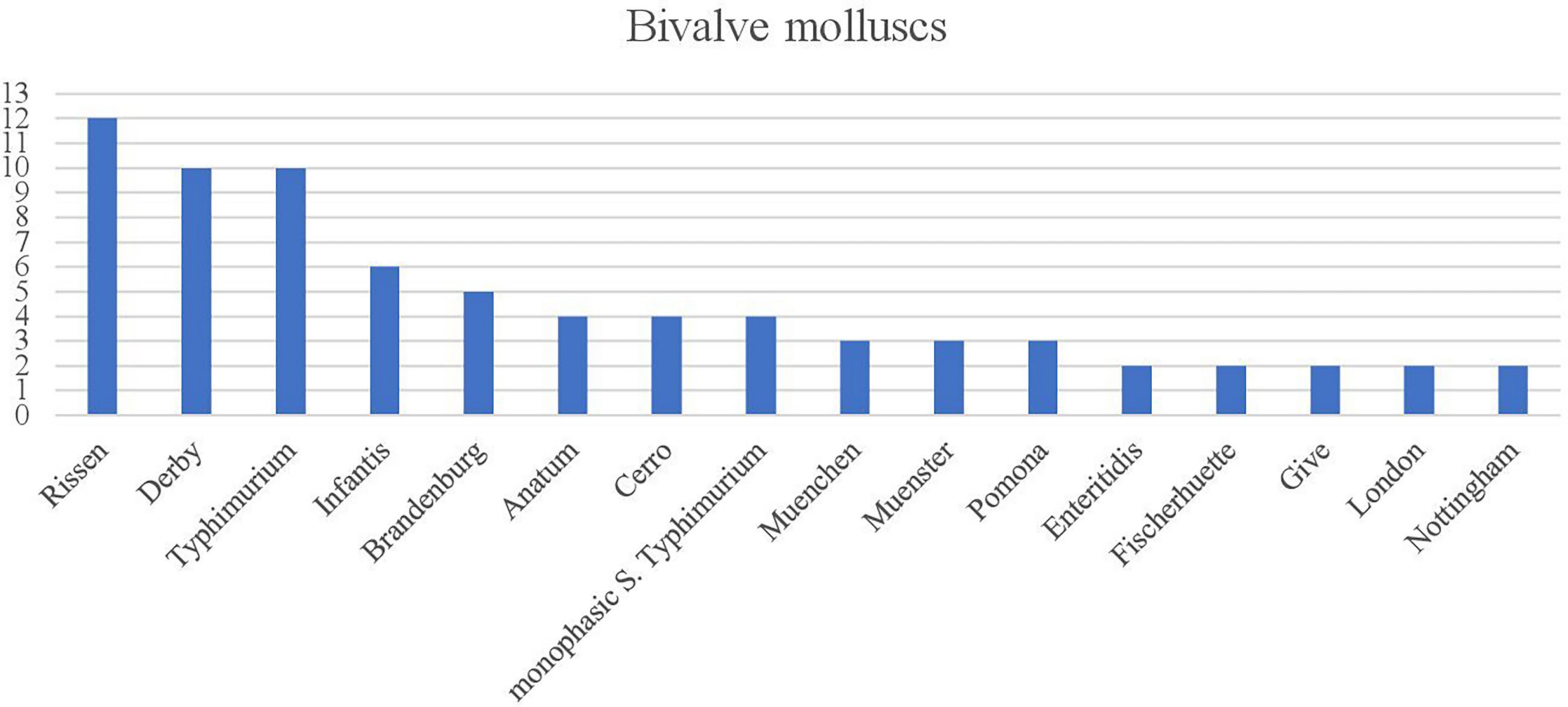
Number of *Salmonella* serovars within the subspecies *enterica* with an occurrence >1 from 2011 to 2021 isolated from Bivalve mollusks from 2011 to 2021.

Results of the serotyping analysis according to the sources are reported in Figures 2, 3, and in the Supplementary materials S3–S6. A total of 326 (99.69%) strains isolated from meat and meat product were assigned to the subspecies *S. enterica* and one to *diarizonae* (0.31%; Supplementary Table 3). *S.* Infantis was the most frequently detected (*N =* 125, 38.23%), followed by *S.* Derby (*N =* 34, 10.40%), *S*. Typhimurium (*N =* 27, 8.26%), and monophasic *S*. Typhimurium (*N =* 17, 5.20%; Supplementary Table 3). As regards the origin, the most frequently detected serovars were *S.* Infantis (*N =* 105, 80.15%) in Broilers, *S*. Typhimurium (*N =* 25, 22.94%) and *S.* Derby (*N =* 24, 22.02%) in pork meat, monophasic *S*. Typhimurium in bovine meat (*N =* 6, 20.69%) and *S.* Newport (*N =* 4, 23.53%) in turkey meat (Figure 2).

By comparing the two sampling periods a significant increase in the prevalence of *S.* Give (2011–2015: 1 vs. 2016–2021: 7) and a significant decrease of *S*. Typhimurium (2011–2015: 21 vs. 2016–2021: 4) was recorded in pork meat (*p* <0.05). In the same period, a significant increase was also observed in the prevalence of *S.* Infantis (2011–2015: 10 vs. 2016–2021: 95) in broiler meat (*p* <0.05).

A total of 114 (99.13%) strains isolated from the “Fish and fishery products” were assigned to the subspecies *S. enterica* and one to the subspecies *houtenae* (0.87%; Supplementary Table 4). Within the category of Bivalve mollusks, *S.* Rissen was the most frequently detected serovar (Figure 3).

**Figure 4:**
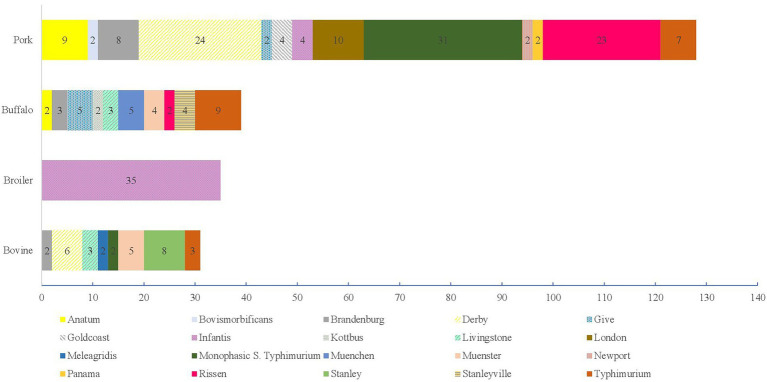
Number of *Salmonella* serovars within the subspecies *enterica* with an occurrence >1 from 2011 to 2021 isolated from Carcasses from 2011 to 2021.

The isolates from carcasses were assigned to the subspecies: *enterica* (*N =* 275, 97.52%), *diarizonae* (*N =* 3, 1.06%), *salamae* (*N =* 3, 1.06%) and *houtenae* (*N =* 1, 0.35%; Supplementary Table 7). *S.* Infantis was the most frequently detected (*N =* 41, 14.91%), followed by monophasic *S*. Typhimurium (*N =* 40, 14.55%), *S.* Derby (*N =* 31, 11.27%), and *S.* Rissen (*N =* 25, 9.09%; Supplementary Table 7). As regards the origin, the most frequently detected serovars were Monophasic *S*. Typhimurium (*N =* 31, 22.14%), *S.* Derby (*N =* 24, 17.14%) and *S.* Rissen (*N =* 23, 16.43%) in pork, *S*. Typhimurium (*N =* 9, 18.00%) in buffalo, *S.* Infantis (*N =* 35, 83.33%) in broilers and *S.* Stanley in bovine (*N =* 8, 21.62%; Figure 4).

## Discussion

In Europe, salmonellosis is one of the most commonly reported foodborne gastrointestinal infections in humans and its trend has been stable from 2011 to 2019 [EFSA and ECDC (European Food Safety Authority and European Centre for Disease Prevention and Control), 2021]. However, compared to previous years, a lower number of confirmed cases was reported in 2020, which is likely due to the COVID-19 pandemic [EFSA and ECDC (European Food Safety Authority and European Centre for Disease Prevention and Control), 2021]. Within this context, the collection of updated data on the circulation of *Salmonella* in foods is necessary for the implementation of more rigorous preventive and control measures.

To our knowledge, this is the first study that provides a complete overview of the occurrence of *Salmonella* spp. in food of animal origin and carcasses between 2011 and 2021 in Italy.

The present work presents results pertinent to: (i) all the analyzes performed in the context of official controls, and (ii) all samples provided to the Zooprophylactic Institute of Southern Italy by private or public enterprises during own-check sampling between 2011–2021 in the Campania and Calabria regions.

In the present study, a comparison of the years 2011–2015 and 2016–2021, an increasing significative trend in the occurrence of positive samples has been observed. Interestingly, the results of the present work showed opposite behaviors compared with those obtained in the years 2002–2013 by Mancin et al. (2018) in which a decrease in the number of *Salmonella* isolation from non-human sources was observed in Italy. In Europe, to our knowledge, data on the occurrence of *Salmonella* in foods between 2011 and 2015 are sparse and have not been aggregated, making it difficult to compare the data present in literature with those of the present work. By contrast, comparing the number of positive food samples recorded in the present study between 2016 and 2020 (5.13%; Supplementary Table 1) with the ones provided by EFSA and ECDC (European Food Safety Authority and European Centre for Disease Prevention and Control) (2021) (positive food samples between 2016 and 2020 = 2.00%), a higher percentage has been observed here. Differences between different countries may be related to the sampling strategies which, according to the Reg. (EC) 2017/625, are risk-based (Focker et al., 2022). Moreover, the study showed a higher prevalence of *Salmonella* positive samples in the Campania region, confirming that *Salmonella* prevalence may also vary between nearby geographical locations within the same country (Ferrari et al., 2019). Although this is speculative, the observation of these differences between the two regions may be due to the higher animal number and livestock density in Campania (ISTAT, 2022), which could influence the spread of the pathogen in the environment.

*Salmonella* was most often observed in food categories of meat origin. Meat can become contaminated with *Salmonella* throughout the entire production chain due to poor hygienic conditions and practices adopted by workers at various stages of processing (Hussain et al., 2020). Comparing the results of the present work with the available data provided by the European food safety authority and the European Center for Disease Prevention and Control [EFSA and ECDC (European Food Safety Authority and European Centre for Disease Prevention and Control), 2021 = 2.30%], the prevalence of meat-positive samples recorded here (13.41%) was higher than the European average. However, the results of the present study are in line with those reported in a previous Italian study, also performed in the Campania region (12%, Carraturo et al., 2016). The highest proportion of *Salmonella-*positive samples was reported for poultry meat, which is a recognized important source of this pathogen for humans (Kalaba et al., 2017). The same picture was reported in European reports [EFSA and ECDC (European Food Safety Authority and European Centre for Disease Prevention and Control), 2021] and by Carraturo et al. (2016) in Italy. For broiler meat, high levels of *Salmonella* positive samples were also recorded outside the European region (China = 36.7%, Yang et al., 2020; Mexico = 18.00%, Regalado-Pineda et al., 2020).

High levels of *Salmonella*-positive samples (6.01%) were also found in the “fishery and fishery products.” Samples of this latter group mainly belonged to bivalve mollusks which, due to their filter-feeding activity, may concentrate pathogenic microorganisms and therefore, if not prepared properly, pose food safety risks (Mudadu et al., 2022). The results of the present work are in contrast to those reported in Europe by EFSA and ECDC for fishery and fishery products [1.55%, EFSA and ECDC (European Food Safety Authority and European Centre for Disease Prevention and Control), 2021] and in the Sardinia region (Italy) by Mudadu et al. (2022) for Bivalve mollusks (0.58%).

Although the number of seed samples was limited compared to other food categories, the percentage of *Salmonella*-positive samples observed in the present study (14.89%) is of particular concern since their consumption is increasing. Seeds can become contaminated with enteric pathogens during growth, harvest and storage [EFSA Panel on Biological Hazards (BIOHAZ), 2011]. In 2020, the Member States of the European Union ordered recalls and withdrawals of such products from their markets for the presence of *Salmonella* (RASFF, 2021). In recent years, seeds have been associated with different human salmonellosis worldwide outbreaks (Yin et al., 2020).

As regards caraccas-positive samples, the highest prevalence was observed in broilers (51.85%), buffalo (49.50%), and pork (36.84%). The high level of contamination observed is probably associated with faecal contamination and therefore improper evisceration procedures (Soare et al., 2021).

A lower prevalence of *Salmonella* was recorded in broilers in a recent Belgian study (Zeng et al., 2021) in which the authors evaluated carcass’ contamination in different slaughterhouses (between 2.1 and 17.7%). In addition, while the Belgian study (Zeng et al., 2021) recorded a decreased prevalence after 15 years, in the present study a significantly increased trend was observed.

A higher number of *Salmonella*-contaminated pork carcasses had been reported in a previous Italian study (87.50%, Peruzy et al., 2021) and in Belgium by Biasino et al. (2018) (64%) but a lower prevalence of *Salmonella* had been observed previously in Korea (Choi et al., 2013; 1%) and in Serbia (Mrdovic et al., 2018; 0.29%).

In buffalo, salmonellosis is a widespread disease characterized by severe gastrointestinal lesions (Borriello et al., 2012). It has been previously reported that the meat can be a reservoir for *Salmonella* (Saud et al., 2019), but, to our knowledge, this is the first study that provides information on the occurrence of this pathogen in carcasses.

In the present study *S. enterica* subsp*. enterica* followed by three non-*enterica* subspecies (*diarizonae*, *salamae*, and *houtenae)* were found. Eight out of eleven strains belonging to the non-*enterica* subspecies were found in wild boar sources. In contrast to the studies of Peruzy et al. (2019), La Tela et al. (2021), and Peruzy et al. (2022) in which all *Salmonella* strains isolated from wild boar killed in the Campania region belonged to the subspecies *enterica*, in the present study, the prevalence of non-*enterica* subspecies was slightly higher (57.14%).

During the period under investigation (2011–2021), serotyping identified 79 serovars. *S.* Infantis, *S.* Derby, monophasic *S*. Typhimurium, *S*. Typhimurium, and *S.* Rissen were the most frequently found. *S*. Typhimurium, monophasic *S*. Typhimurium, *S.* Infantis, and *S.* Derby are frequently reported in human cases in Europe [EFSA and ECDC (European Food Safety Authority and European Centre for Disease Prevention and Control), 2021]. Interestingly, *S.* Enteritidis, the most commonly reported serovar from confirmed human cases in Europe since 2011 [EFSA and ECDC (European Food Safety Authority and European Centre for Disease Prevention and Control), 2021] was not frequently detected in the present study, and comparing the period 2011–2015 with 2016–2021, a decreased, although not significative, occurrence was observed (*p* > 0.05). By contrast, *S.* Rissen, frequently detected here, is often associated with human infections in the United States of America and Asia (Silveira et al., 2019), but not in the European Union.

As has already been reported by EFSA and ECDC (European Food Safety Authority and European Centre for Disease Prevention and Control) (2021), *S.* Infantis was strictly related to broiler sources (meat and carcass). In the present study, *S.* Enteritidis was never isolated from these matrices. This result is in contrast with those reported by several studies which confirm poultry products as unquestionably associated with *S.* Enteritidis serovars in Europe (Foley et al., 2011; Cosby et al., 2015; Ferrari et al., 2019). However, nowadays, *S.* Infantis is recognized worldwide as the predominant *Salmonella* serovar in poultry (Kalaba et al., 2017). The increasing prevalence of *S.* Infantis in poultry meat could be a result of the implementation of vaccinations against *S.* Enteritidis and *S*. Typhimurium in recent years (Kalaba et al., 2017). As hypothesized by EFSA and ECDC (European Food Safety Authority and European Centre for Disease Prevention and Control) (2021), the control programe has probably allowed an expansion of other serovars that have found new niches in the poultry industry.

As concerns pork sources, in the present study, the most reported serovars were *S*. Typhimurium and *S.* Derby in meat and meat products and monophasic *S*. Typhimurium, *S.* Derby, and *S.* Rissen in carcasses. The predominance of these latter serovars in the pig production chain in Italy has already been reported by several authors (Bonardi et al., 2016, 2017; Pala et al., 2019; Piras et al., 2019; Chalias et al., 2022). *S.* Derby is known to be associated with pigs and in recent years, it has been increasingly isolated in human cases in Europe [EFSA and ECDC (European Food Safety Authority and European Centre for Disease Prevention and Control), 2021]. While observing *S*. Typhimurium in the present work by comparing the years 2011–2016 with 2016–2021, a significant decrease has been observed (*p* <0.05). This result is in contrast to the findings of other studies which reported an increasing prevalence both in human cases and pork products (Arguello et al., 2013; Van Damme et al., 2018; Piras et al., 2019).

According to the findings of the present work, monophasic *S*. Typhimurium was the dominant serovar in bovine meat and *S.* Stanley in the bovine carcasses. *S.* Stanley is one of the most common serovars associated with human infections in south-east Asia. It has also been present for some years now in the European Union and it is circulating within the European food market [ECDC and EFSA (European Centre for Disease Prevention and Control, European Food Safety Authority), 2014; Aung et al., 2020]. However, *S.* Stanley is usually associated with other animal species and is not as related to bovine carcasses as reported in the present study [ECDC and EFSA (European Centre for Disease Prevention and Control, European Food Safety Authority), 2014; Yang et al., 2019; Aung et al., 2020]. There is a contrast in results between those reported by Wieczorek and Osek (2013) in Poland, in which *S.* Enteritidis and *S.* Schleissheim, and *S.* Dublin were the most predominant serovars in slaughtered cattle and beef and results recently reported by Cetin et al. (2019) in Turkey which frequently isolated *S*. Typhimurium, *S.* Enteritidis and *S.* Newport from Cattle and by Hussain et al. (2020) in which *S.* Enteritidis, *S.* Cholerasuis, *S*. Typhimurium and *S.* Pullorom were frequently found in raw beef in Pakistan.

The dominant serovars found in seafood were *S*. Typhimurium, *S.* Derby, *S.* Rissen, and *S.* Infantis. It is not surprising that *Salmonella* serovar distribution in seafood reflects those found in other animal sources since an aquatic environment may become contaminated by microorganisms introduced *via* animal and human waste during processing and/or production chain preparation (Ferrari et al., 2019).

In conclusion, the data presented here compile an overview of the prevalence and distribution of serovars in the south of Italy. A high occurrence of *Salmonella* spp. in food and in carcasses of food-producing animals was found. Despite the implementation of European and national control strategies against *Salmonella*, results of the present work revealed that the prevalence of this pathogen in southern Italy is still increasing. Moreover, the increase of *S.* Infantis (*p* <0.05) and monophasic *S*. Typhimurium (*p* <0.05) and the decrease of *S*. Typhimurium (*p* <0.05) and *S.* Enteritidis (*p* > 0.05) observed during the period under investigation (2011 to 2021) demonstrate that *Salmonella* serovar distribution has changed. Considering the risk associated with *Salmonella* spp. contamination, more stringent control interventions are needed at primary production and throughout the food supply chain. Moreover, a change in food legislation and *Salmonella* control programmes that focus more on the current predominant serovars would be indispensable.

## Data availability statement

The original contributions presented in the study are included in the article/Supplementary material, further inquiries can be directed to the AB, anna.balestrieri@izsmportici.it.

## Author contributions

YP, AB, and FC: conceptualization. AM: methodology. DC: formal analysis. AM: investigation. AB: data curation. MP and NM: writing—original draft preparation. All authors contributed to the article and approved the submitted version.

## Conflict of interest

The authors declare that the research was conducted in the absence of any commercial or financial relationships that could be construed as a potential conflict of interest.

## Publisher’s note

All claims expressed in this article are solely those of the authors and do not necessarily represent those of their affiliated organizations, or those of the publisher, the editors and the reviewers. Any product that may be evaluated in this article, or claim that may be made by its manufacturer, is not guaranteed or endorsed by the publisher.
