# Supplementary material for: Occurrence and distribution of Salmonella serovars in carcasses and foods in southern Italy: Eleven-year monitoring (2011–2021)
Source: Front Microbiol. 2022 Oct 6;13:1005035. doi: 10.3389/fmicb.2022.1005035 (PMC9582760; doi:10.3389/fmicb.2022.1005035)
Supplement: Supplementary file 2 [file Table_2.DOCX]

S2. Number (N) and percentage (%) of *Salmonella* serovars within the subspecies *enterica* isolated from the 13,228 samples collected grouped by years (2011-2015 *vs* 2016-2021)

|  |  |  | 2011-2015 | | 2016-2021 | |
| --- | --- | --- | --- | --- | --- | --- |
| Species | Subspecies | Serovars | N. | % | N. | % |
| *S. enterica* | *enterica* | Agama | 1 | 0.45 |  |  |
|  |  | Agbeni | 1 | 0.45 | 1 | 0.20 |
|  |  | Agona | 1 | 0.45 | 6 | 1.22 |
|  |  | Anatum | 4 | 1.80 | 15 | 3.04 |
|  |  | Bareilly | 1 | 0.45 |  |  |
|  |  | Bispebjerg | 1 | 0.45 |  |  |
|  |  | Blockley |  |  | 2 | 0.41 |
|  |  | Bovismorbificans | 1 | 0.45 | 4 | 0.81 |
|  |  | Brandenburg | 3 | 1.35 | 20 | 4.06 |
|  |  | Bredeney | 5 | 2.25 | 6 | 1.22 |
|  |  | Cannstatt | 1 | 0.45 |  |  |
|  |  | Carno |  |  | 2 | 0.41 |
|  |  | Cerro | 1 | 0.45 | 3 | 0.61 |
|  |  | Coeln | 1 | 0.45 | 1 | 0.20 |
|  |  | Derby | 28 | 12.61 | 49 | 9.94 |
|  |  | Edinburg | 1 | 0.45 |  |  |
|  |  | Eko | 1 | 0.45 |  |  |
|  |  | Enteritidis | 4 | 1.80 | 4 | 0.81 |
|  |  | Fischerhuette |  |  | 2 | 0.41 |
|  |  | Galil | 1 | 0.45 |  |  |
|  |  | Gallinarum biovar Pullorum |  |  | 1 | 0.20 |
|  |  | Give | 6 | 2.70 | 13 | 2.64 |
|  |  | Goldcoast |  |  | 6 | 1.22 |
|  |  | Hadar | 1 | 0.45 |  |  |
|  |  | Hato | 1 | 0.45 |  |  |
|  |  | Havana |  |  | 1 | 0.20 |
|  |  | Heidelberg | 1 | 0.45 |  |  |
|  |  | Hermannswerder | 1 | 0.45 |  |  |
|  |  | Hisingen | 1 | 0.45 |  |  |
|  |  | Hvittingfoss | 1 | 0.45 |  |  |
|  |  | Infantis | 30 | 13.51 | 147 | 29.82 |
|  |  | Inganda | 1 | 0.45 |  |  |
|  |  | Kapemba | 3 | 1.35 |  |  |
|  |  | Kasenyi |  |  | 2 | 0.41 |
|  |  | Kentucky | 1 | 0.45 | 1 | 0.20 |
|  |  | Kottbus | 1 | 0.45 | 3 | 0.61 |
|  |  | Litchfield |  |  | 1 | 0.20 |
|  |  | Livingstone | 9 | 4.05 | 14 | 2.84 |
|  |  | London | 7 | 3.15 | 19 | 3.85 |
|  |  | Manchester |  |  | 1 | 0.20 |
|  |  | Manhattan | 1 | 0.45 |  |  |
|  |  | Mbandaka |  |  | 1 | 0.20 |
|  |  | Meleagridis | 1 | 0.45 | 3 | 0.61 |
|  |  | Mishmarhaemek |  |  | 1 | 0.20 |
|  |  | Monophasic *S.* Typhimurium | 7 | 3.15 | 56 | 11.36 |
|  |  | Muenchen | 3 | 1.35 | 7 | 1.42 |
|  |  | Muenster | 7 | 3.15 | 7 | 1.42 |
|  |  | Napoli | 2 | 0.90 | 1 | 0.20 |
|  |  | Newport | 7 | 3.15 | 4 | 0.81 |
|  |  | Nottingham |  |  | 4 | 0.81 |
|  |  | Ohio | 1 | 0.45 |  |  |
|  |  | Panama |  |  | 4 | 0.81 |
|  |  | Paratyphi b | 5 | 2.25 |  |  |
|  |  | Pomona |  |  | 3 | 0.61 |
|  |  | Reading | 1 | 0.45 |  |  |
|  |  | Rissen | 13 | 5.86 | 34 | 6.90 |
|  |  | Saintpaul | 3 | 1.35 |  |  |
|  |  | Salford |  |  | 1 | 0.20 |
|  |  | Sandiego |  |  | 1 | 0.20 |
|  |  | Schleissheim | 1 | 0.45 |  |  |
|  |  | Senftenberg | 2 | 0.90 |  |  |
|  |  | Soerenga |  |  | 1 | 0.20 |
|  |  | Stanley |  |  | 11 | 2.23 |
|  |  | Stanleyville | 4 | 1.80 | 3 | 0.61 |
|  |  | Szentes |  |  | 1 | 0.20 |
|  |  | Tennessee |  |  | 1 | 0.20 |
|  |  | Thompson | 2 | 0.90 | 1 | 0.20 |
|  |  | Toulon | 1 | 0.45 |  |  |
|  |  | Tounouma |  |  | 1 | 0.20 |
|  |  | Typhimurium | 35 | 15.77 | 19 | 3.85 |
|  |  | Uganda |  |  | 1 | 0.20 |
|  |  | Umbilo |  |  | 1 | 0.20 |
|  |  | Veneziana | 1 | 0.45 |  |  |
|  |  | Virchow | 1 | 0.45 |  |  |
|  |  | Weltevreden | 1 | 0.45 | 1 | 0.20 |
|  |  | Westhampton | 1 | 0.45 |  |  |
|  |  | Wil | 1 | 0.45 |  |  |
|  |  | Winston |  |  | 1 | 0.20 |
|  |  | Worthington | 1 | 0.45 |  |  |
