# Supplementary material for: Occurrence and distribution of Salmonella serovars in carcasses and foods in southern Italy: Eleven-year monitoring (2011–2021)
Source: Front Microbiol. 2022 Oct 6;13:1005035. doi: 10.3389/fmicb.2022.1005035 (PMC9582760; doi:10.3389/fmicb.2022.1005035)
Supplement: Supplementary file 3 [file Table_3.DOCX]

S3. Number of *Salmonella* serovars/antigenic formulae isolated from 2011 to 2021 from “Meat and Meat Products” grouped by animal species. N.I. No information on the animal speices or serovars/antigenic formulae. MMP-B&T: Mixed meat products of Broiler and Turkey. MMP-B&Bo: Mixed meat products of Broiler and Bovine. MMP-B&T&P: Mixed meat products of Broiler, Turkey and Pork.

|  |  |  |  | Meat and Meat Products | | | | | | | | | | |
| --- | --- | --- | --- | --- | --- | --- | --- | --- | --- | --- | --- | --- | --- | --- |
| Species | Subspecies |  | Serovar/antigenic formulae | Bovine | Buffalo | Pork | Broiler | Turkey | Wild Boar | MMP-B&T | MMP- B&Bo | MMP-B&T&P | N.I. | Tot. |
| *S. enterica* | *enterica* |  | Agama |  |  |  |  |  |  |  |  |  | 1 | 1 |
|  |  |  | Agona | 3 |  |  | 1 | 1 |  |  |  |  |  | 5 |
|  |  |  | Anatum | 1 |  | 3 |  |  |  |  |  |  |  | 4 |
|  |  |  | Blockley |  |  |  | 1 |  |  |  |  | 1 |  | 2 |
|  |  |  | Bovismorbificans |  |  |  | 1 |  |  |  |  |  |  | 1 |
|  |  |  | Brandenburg | 1 |  | 3 |  |  | 1 |  |  |  |  | 5 |
|  |  |  | Bredeney | 1 |  | 4 | 2 |  |  | 1 |  |  |  | 8 |
|  |  |  | Carno | 1 |  |  |  |  |  |  |  |  |  | 1 |
|  |  |  | Coeln |  |  |  |  |  |  | 1 |  |  | 1 | 2 |
|  |  |  | Derby | 4 |  | 24 |  | 1 |  | 3 |  | 1 | 1 | 34 |
|  |  |  | Edinburg |  |  | 1 |  |  |  |  |  |  |  | 1 |
|  |  |  | Eko | 1 |  |  |  |  |  |  |  |  |  | 1 |
|  |  |  | Enteritidis | 1 |  |  |  |  |  |  |  |  |  | 1 |
|  |  |  | Give |  |  | 8 | 2 |  |  |  |  |  |  | 10 |
|  |  |  | Goldcoast |  |  | 1 |  |  |  |  |  |  |  | 1 |
|  |  |  | Hadar |  |  |  |  |  |  | 1 |  |  |  | 1 |
|  |  |  | Heidelberg |  |  |  | 1 |  |  |  |  |  |  | 1 |
|  |  |  | Infantis | 3 |  | 1 | 105 | 2 |  | 11 | 1 |  | 2 | 125 |
|  |  |  | Kapemba | 2 |  | 1 |  |  |  |  |  |  |  | 3 |
|  |  |  | Kottbus |  | 1 |  |  | 1 |  |  |  |  |  | 2 |
|  |  |  | Livingstone |  |  | 8 | 1 |  | 1 |  |  |  |  | 10 |
|  |  |  | London | 1 |  | 1 | 6 |  |  | 1 |  |  | 1 | 10 |
|  |  |  | Mishmarhaemek |  |  | 1 |  |  |  |  |  |  |  | 1 |
|  |  |  | monophasic S. Typhimurium | 6 |  | 8 | 1 |  |  | 2 |  |  |  | 17 |
|  |  |  | Muenchen |  |  | 1 |  |  |  |  |  |  |  | 1 |
|  |  |  | Muenster | 1 |  |  |  |  |  |  |  |  |  | 1 |
|  |  |  | Newport |  |  |  | 2 | 4 |  |  |  |  |  | 6 |
|  |  |  | Paratyphi b |  |  |  | 1 | 2 |  |  |  |  |  | 3 |
|  |  |  | Rissen | 1 |  | 7 | 1 |  |  |  |  |  |  | 9 |
|  |  |  | Saintpaul |  |  |  | 1 | 2 |  |  |  |  |  | 3 |
|  |  |  | Sandiego |  |  |  |  |  |  | 1 |  |  |  | 1 |
|  |  |  | Senftenberg |  |  |  |  |  |  |  |  |  | 2 | 2 |
|  |  |  | Stanley | 1 |  |  |  |  |  |  |  |  |  | 1 |
|  |  |  | Stanleyville |  | 1 |  |  |  |  |  |  |  |  | 1 |
|  |  |  | Tennessee |  |  |  | 1 |  |  |  |  |  |  | 1 |
|  |  |  | Thompson |  |  |  | 2 |  | 1 |  |  |  |  | 3 |
|  |  |  | Typhimurium |  |  | 25 |  |  |  |  |  |  | 2 | 27 |
|  |  |  | Wil |  |  |  |  |  |  |  |  |  | 1 | 1 |
|  |  |  | N. I. | 1 |  | 12 | 2 | 4 |  |  |  |  |  | 19 |
|  | diarizonae IIIb |  | 38:l,v:z35 |  |  |  |  |  | 1 |  |  |  |  | 1 |
| Tot. |  |  |  | 29 | 2 | 109 | 131 | 17 | 4 | 21 | 1 | 2 | 11 | 327 |
