# Supplementary material for: Occurrence and distribution of Salmonella serovars in carcasses and foods in southern Italy: Eleven-year monitoring (2011–2021)
Source: Front Microbiol. 2022 Oct 6;13:1005035. doi: 10.3389/fmicb.2022.1005035 (PMC9582760; doi:10.3389/fmicb.2022.1005035)
Supplement: Supplementary file 4 [file Table_4.DOCX]

S4. Number of *Salmonella* serovars/antigenic formulae isolated from 2011 to 2021 from “Fish and fishery products” N.I. No information on serovars/antigenic formulae.

|  |  |  | Fish and fishery products | | | | | Tot |
| --- | --- | --- | --- | --- | --- | --- | --- | --- |
| Species | Subspecies | Serovar | Bivalve molluscs | Cephalopod molluscs | Molluscs | Crustaceans | Others |  |
| *S. enterica* | *enterica* | Agbeni | 1 |  |  |  |  | 1 |
|  |  | Agona | 1 |  |  |  |  | 1 |
|  |  | Anatum | 4 |  |  |  |  | 4 |
|  |  | Brandenburg | 5 |  |  |  |  | 5 |
|  |  | Bredeney | 1 |  |  |  |  | 1 |
|  |  | Cerro | 4 |  |  |  |  | 4 |
|  |  | Derby | 10 |  | 2 |  |  | 12 |
|  |  | Enteritidis | 2 |  |  |  |  | 2 |
|  |  | Fischerhuette | 2 |  |  |  |  | 2 |
|  |  | Give | 2 |  |  |  |  | 2 |
|  |  | Hato | 1 |  |  |  |  | 1 |
|  |  | Havana | 1 |  |  |  |  | 1 |
|  |  | Infantis | 6 |  | 4 |  |  | 10 |
|  |  | Kentucky | 1 |  |  |  |  | 1 |
|  |  | Litchfield | 1 |  |  |  |  | 1 |
|  |  | Livingstone | 1 |  | 1 | 1 |  | 3 |
|  |  | London | 2 |  | 1 |  |  | 3 |
|  |  | Manhattan |  |  | 1 |  |  | 1 |
|  |  | Mbandaka | 1 |  |  |  |  | 1 |
|  |  | Meleagridis |  |  | 1 |  |  | 1 |
|  |  | monophasic S. Typhimurium | 4 |  |  |  |  | 4 |
|  |  | Muenchen | 3 |  |  |  |  | 3 |
|  |  | Muenster | 3 |  |  |  |  | 3 |
|  |  | Newport |  | 1 |  |  |  | 1 |
|  |  | Nottingham | 2 |  |  |  |  | 2 |
|  |  | Ohio | 1 |  |  |  |  | 1 |
|  |  | Panama | 1 |  |  |  |  | 1 |
|  |  | Paratyphi b | 1 |  |  |  |  | 1 |
|  |  | Pomona | 3 |  |  |  |  | 3 |
|  |  | Reading | 1 |  |  |  |  | 1 |
|  |  | Rissen | 12 |  |  |  |  | 12 |
|  |  | Schleissheim |  | 1 |  |  |  | 1 |
|  |  | Stanley | 1 |  |  |  |  | 1 |
|  |  | Stanleyville | 1 |  |  |  |  | 1 |
|  |  | Typhimurium | 10 |  | 3 |  |  | 13 |
|  |  | Virchow |  |  | 1 |  |  | 1 |
|  |  | Weltevreden |  | 1 |  |  |  | 1 |
|  |  | Westhampton | 1 |  |  |  |  | 1 |
|  |  | Worthington | 1 |  |  |  |  | 1 |
|  |  | N.I. | 5 |  |  |  |  | 5 |
|  | *houtenae* | N.I. |  |  |  |  | 1 | 1 |
| Tot. |  |  | 96 | 3 | 14 | 1 | 1 | 115 |
