# Supplementary material for: Occurrence and distribution of Salmonella serovars in carcasses and foods in southern Italy: Eleven-year monitoring (2011–2021)
Source: Front Microbiol. 2022 Oct 6;13:1005035. doi: 10.3389/fmicb.2022.1005035 (PMC9582760; doi:10.3389/fmicb.2022.1005035)
Supplement: Supplementary file 5 [file Table_5.DOCX]

S5. Number of *Salmonella* serovars isolated from 2011 to 2021 from “Milk and milk products”

|  |  |  | Milk and milk products | | | Tot |
| --- | --- | --- | --- | --- | --- | --- |
| Species | Subspecies | Serovar | Processed Milk | Raw Milk | Dairy products |  |
| *S. enterica* | *enterica* | Agbeni |  |  | 1 | 1 |
|  |  | Bovismorbificans |  | 1 |  | 1 |
|  |  | Hisingen |  |  | 1 | 1 |
|  |  | London |  | 2 |  | 2 |
|  |  | monophasic S. Typhimurium | 1 |  | 1 | 2 |
|  |  | Muenchen |  | 1 |  | 1 |
|  |  | Stanleyville |  | 1 |  | 1 |
|  |  | Toulon |  |  | 1 | 1 |
|  |  | Typhimurium |  | 1 |  | 1 |
|  |  | N.I. |  | 3 |  | 3 |
| *S. enterica* | *diarizonae* |  |  | 1 | 1 | 2 |
| Tot. |  |  | 1 | 10 | 5 | 16 |

N.I. No information on serovars
