# Supplementary material for: Occurrence and distribution of Salmonella serovars in carcasses and foods in southern Italy: Eleven-year monitoring (2011–2021)
Source: Front Microbiol. 2022 Oct 6;13:1005035. doi: 10.3389/fmicb.2022.1005035 (PMC9582760; doi:10.3389/fmicb.2022.1005035)
Supplement: Supplementary file 6 [file Table_6.DOCX]

S6 Number of *Salmonella* serovars isolated from 2011 to 2021 from Eggs and egg products, Bakery products, cereals, Fruit and vegetables and juices, Seeds (sprouted seeds) , Spices and herbs and other processed food products and prepared dishes.

|  |  |  | Eggs and egg products | Bakery products | Cereals | Fruits and vegetables and juices | Seeds (sprouted seeds) | Spices and herbs | Other processed food products and prepared dishes | Tot. |
| --- | --- | --- | --- | --- | --- | --- | --- | --- | --- | --- |
| Species | Subspecies | Serovar/Antigenic Formulae |  |  |  |  |  |  |  |  |
| *S. enterica* | *enterica* | Kasenyi |  |  |  | 1 |  |  |  | 1 |
|  |  | Napoli |  |  |  | 1 |  | 1 |  | 2 |
|  |  | Nottingham |  |  |  | 1 |  |  |  | 1 |
|  |  | Veneziana |  |  |  | 1 |  |  |  | 1 |
|  |  | Winston |  |  |  | 1 |  |  |  | 1 |
|  |  | Bareilly |  |  |  |  | 1 |  |  | 1 |
|  |  | Bispebjerg |  |  |  |  | 1 |  |  | 1 |
|  |  | Cannstatt |  |  |  |  | 1 |  |  | 1 |
|  |  | Galil |  |  |  |  | 1 |  |  | 1 |
|  |  | Hvittingfoss |  |  |  |  | 1 |  |  | 1 |
|  |  | Inganda |  |  |  |  | 1 |  |  | 1 |
|  |  | Livingstone |  |  |  |  | 1 |  |  | 1 |
|  |  | Meleagridis |  |  |  |  | 1 |  |  | 1 |
|  |  | Rissen |  |  |  |  | 1 |  |  | 1 |
|  |  | Salford |  |  |  |  | 1 |  |  | 1 |
|  |  | Soerenga |  |  |  |  | 1 |  |  | 1 |
|  |  | Weltevreden |  |  |  |  |  |  | 1 | 1 |
|  |  | Enteritidis | 1 | 1 |  |  |  |  |  | 2 |
|  |  | Gallinarum biovar Pullorum | 1 |  |  |  |  |  |  | 1 |
|  |  | Hermannswerder | 1 |  |  |  |  |  |  | 1 |
|  |  | Infantis | 1 |  |  |  |  |  |  | 1 |
|  |  | Kentucky | 1 |  |  |  |  |  |  | 1 |
|  |  | Livingstone | 2 |  | 1 |  |  |  |  | 3 |
|  |  | Manchester | 1 |  |  |  |  |  |  | 1 |
|  |  | N.I. |  |  |  | 1 | 2 |  | 1 | 4 |
| *S. enterica* | *diarizonae* | P:38:l,v:- |  |  |  |  | 1 |  |  | 1 |
| Tot. |  |  | 8 | 1 | 1 | 6 | 14 | 1 | 2 | 33 |

N.I. No information on serovars
