# Supplementary material for: Occurrence and distribution of Salmonella serovars in carcasses and foods in southern Italy: Eleven-year monitoring (2011–2021)
Source: Front Microbiol. 2022 Oct 6;13:1005035. doi: 10.3389/fmicb.2022.1005035 (PMC9582760; doi:10.3389/fmicb.2022.1005035)
Supplement: Supplementary file 7 [file Table_7.DOCX]

S7. Number of *Salmonella* serovars/antigenic formulae isolated from 2011 to 2021 on carcasses of Bovine, Buffalo, Goat, Ovine, Pork, Broilers and Wild Boars. No information on serovars/antigenic formulae.

|  |  |  | Carcass | | | | | | | |
| --- | --- | --- | --- | --- | --- | --- | --- | --- | --- | --- |
| Species | Subspecies | Serovar /antigenic Formulae | Bovine | Buffalo | Goat | Ovine | Pork | Broilers | Wild Boar | Tot. |
| *S. enterica* | *enterica* | Agona | 1 |  |  |  |  |  |  | 1 |
|  |  | Anatum |  | 2 |  |  | 9 |  |  | 11 |
|  |  | Bovismorbificans | 1 |  |  |  | 2 |  |  | 3 |
|  |  | Brandenburg | 2 | 3 |  |  | 8 |  |  | 13 |
|  |  | Bredeney | 1 |  |  |  | 1 |  |  | 2 |
|  |  | Carno |  | 1 |  |  |  |  |  | 1 |
|  |  | Derby | 6 | 1 |  |  | 24 |  |  | 31 |
|  |  | Enteritidis |  |  | 1 | 1 |  | 1 |  | 3 |
|  |  | Give |  | 5 |  |  | 2 |  |  | 7 |
|  |  | Goldcoast |  | 1 |  |  | 4 |  |  | 5 |
|  |  | Infantis | 1 |  |  |  | 4 | 35 | 1 | 41 |
|  |  | Kasenyi |  | 1 |  |  |  |  |  | 1 |
|  |  | Kottbus |  | 2 |  |  |  |  |  | 2 |
|  |  | Livingstone | 3 | 3 |  |  |  |  |  | 6 |
|  |  | London |  | 1 |  |  | 10 |  |  | 11 |
|  |  | Meleagridis | 2 |  |  |  |  |  |  | 2 |
|  |  | Monophasic *S.* Typhimurium | 2 |  |  |  | 31 |  |  | 33 |
|  |  | Muenchen |  | 5 |  |  |  |  |  | 5 |
|  |  | Muenster | 5 | 4 |  |  | 1 |  |  | 10 |
|  |  | Napoli |  |  |  |  |  |  | 1 | 1 |
|  |  | Newport |  | 1 |  |  | 2 | 1 |  | 4 |
|  |  | Nottingham |  |  |  |  | 1 |  |  | 1 |
|  |  | Panama | 1 |  |  |  | 2 |  |  | 3 |
|  |  | Paratyphi b |  | 1 |  |  |  |  |  | 1 |
|  |  | Rissen |  | 2 |  |  | 23 |  |  | 25 |
|  |  | Stanley | 8 | 1 |  |  |  |  |  | 9 |
|  |  | Stanleyville |  | 4 |  |  |  |  |  | 4 |
|  |  | Szentes |  | 1 |  |  |  |  |  | 1 |
|  |  | Tounouma |  | 1 |  |  |  |  |  | 1 |
|  |  | Typhimurium | 3 | 9 |  |  | 7 |  | 1 | 20 |
|  |  | Uganda |  |  |  |  | 1 |  |  | 1 |
|  |  | Umbilo |  | 1 |  |  |  |  |  | 1 |
|  |  | N.I. | 1 |  |  | 1 | 8 | 5 |  | 15 |
|  | *salamae* | 41:z:1,5 |  |  |  |  |  |  | 3 | 3 |
|  | *diarizonae* | 50:r:1,5 |  |  |  |  |  |  | 2 | 2 |
|  |  | 65:-:z |  |  |  |  |  |  | 1 | 1 |
|  | *houtenae* | 38:z4z23:- |  |  |  |  |  |  | 1 | 1 |
|  | Tot. |  | 37 | 50 | 1 | 2 | 140 | 42 | 10 | 282 |
